# Towards an Understanding of the Interactions between Freshwater Inflows and Phytoplankton Communities in a Subtropical Estuary in the Gulf of Mexico

**DOI:** 10.1371/journal.pone.0130931

**Published:** 2015-07-02

**Authors:** Samuel Dorado, Tyra Booe, Jamie Steichen, Allison S. McInnes, Rachel Windham, Alicia Shepard, Allyson E. B. Lucchese, Hannah Preischel, James L. Pinckney, Stephen E. Davis, Daniel L. Roelke, Antonietta Quigg

**Affiliations:** 1 Department of Marine Biology, Texas A&M University at Galveston, Galveston, Texas, 77553, United States of America; 2 Department of Oceanography, Texas A&M University, College Station, Texas, 77843, United States of America; 3 Biological Sciences, University of South Carolina, Columbia, South Carolina, 29208, United States of America; 4 Marine Science Program, University of South Carolina, Columbia, South Carolina, 29208, United States of America; 5 Department of Wildlife and Fisheries Sciences, Texas A&M University, College Station, Texas, 77843, United States of America; 6 Everglades Foundation, Palmetto Bay, Florida, 33157, United States of America; University of Connecticut, UNITED STATES

## Abstract

Subtropical estuaries worldwide face increased pressure on their ecosystem health and services due to increasing human population growth and associated land use/land cover changes, expansion of ports, and climate change. We investigated freshwater inflows (river discharge) and the physico-chemical characteristics of Galveston Bay (Texas, USA) as mechanisms driving variability in phytoplankton biomass and community composition between February 2008 and December 2009. Results of multivariate analyses (hierarchical cluster analysis, PERMANOVA, Mantel test, and nMDS ordination coupled to environmental vector fitting) revealed that temporal and spatial differences in phytoplankton community structure correlate to differences in hydrographic and water quality parameters. Spatially, phytoplankton biomass and community composition responded to nutrient loading from the San Jacinto River in the northwest region of the bay (consistent with nutrient limitation) while hydraulic displacement (and perhaps other processes) resulted in overall lower biomass in the Trinity River delta (northeast region). The influence of inflows on phytoplankton diminished along a north to south gradient in the bay. Temporally, temperature and variables associated with freshwater inflow (discharge volume, salinity, inorganic nitrogen and phosphorus concentrations) were major influences on phytoplankton dynamics. Dissolved inorganic nitrogen: phosphorus (DIN:DIP) ratios suggest that phytoplankton communities will be predominately nitrogen limited. Diatoms dominated during periods of moderate to high freshwater inflows in winter/spring and were more abundant in the upper bay while cyanobacteria dominated during summer/fall when inflow was low. Given the differential influences of freshwater inflow on the phytoplankton communities of Galveston Bay, alterations upstream (magnitude, timing, frequency) will likely have a profound effect on downstream ecological processes and corresponding ecosystem services.

## Introduction

The health and welfare of aquatic and marine ecosystems has changed significantly over the last 100–200 years [1–6]. Substantial milestones in the principles and objectives governing freshwater management in the US include, but are not limited to, the establishment of the Army Corps of Engineers (in 1802; http://www.usace.army.mil/) and the Reclamation Service (in 1902 http://www.usbr.gov/) which have created water efficiencies for irrigation, navigation, and flood protection (e.g., the Hoover Dam). Important 20^th^ century environmental management legislation included the National Environmental Policy Act (1969), the Clean Water Act (1972) and the Endangered Species Act (1973), which deal specifically with regulating impacts to US waterways (www.epa.gov). Similar governance and protection of freshwater resources has been applied worldwide (see United Nations Environmental Programme; www.unep.org/). In order to inform modern management strategies and regulation procedures scientists are challenged to define processes within target systems, specifically estuaries and coasts, where landscapes are shifting in response to human activities and climate change.

Changes in land use, riverine diversions and construction (e.g., levies, dams) have altered both quantities and ratios of nutrients and sediment loading into our rivers, estuaries and coasts. Significant hypoxia and algal (some harmful) blooms observed on the Louisiana continental shelf in the Gulf of Mexico are argued to be the result of nutrient enrichment in the Mississippi River, one of the world’s longest rivers and the largest drainage system in North America [7–9]. Similar observations have been made for other highly modified complexes including the Nile River in Africa [10], the Yangtze River in Asia [11], the Baltic Sea in Europe [12] and others [1,3–5,13].

Coastal development is occurring at an unprecedented pace such that concerns about the susceptibility and resilience of these ecotones need to be addressed. Desalination processes, for example, are required to produce freshwater for human consumption in many rapidly developing but arid countries. These processes however, result in the salinization of coastal waters, which has been linked to increased frequency of algal blooms [14–15]. When toxins are present, contaminated water supplies can no longer be used to support human requirements. Shellfish and finfish populations may be subjected to these toxins resulting in fish kill events [16] and in some cases overfishing is also occurring, making it difficult to separate one phenomenon from the other [17]. Further, shipping traffic and the consequent discharge of ballast waters into receiving ports has led to an increase in propagule pressure and concurrently an increase in the appearance of invasive species in many coastal systems [18–19].

Identifying relationships between phytoplankton and freshwater inflows (river discharge into estuaries) is important for predicting ecological impacts resulting from urbanization and industrialization upstream as well as climate change and sea level rise associated with coastal processes downstream [20–21]. Phytoplankton populations are especially sensitive to changes in water chemistry and nutrient regimes [1,6,13,22–23]. Nutrient loading driven by inflow events temper primary production and alter phytoplankton community composition [1,2,4–6,22–24]. When supplied in the appropriate concentrations and ratios, nutrients contribute positively to estuarine water quality and resident primary producers [24–26]. While nitrogen is primarily considered, phosphate stimulation of phytoplankton is also observed [22,27–30]. Variability of phytoplankton responses (biomass, community composition, turnover rates, timing and magnitude of blooms) in turn influences higher trophic levels that depend on them for the assimilation of organic matter. While much of the work to date has been done in temperate ecosystems such as Chesapeake Bay [28,31] and San Francisco Bay [32], less is understood about interactions between freshwater inflows and phytoplankton dynamics in subtropical estuaries.

As the second largest estuary in the northern Gulf of Mexico (~1554 km^2^) and seventh largest in the United States, Galveston Bay, receives ~60% of the urban and industrial waste water (treated and untreated) of Texas [33]. The Galveston Bay watershed, which includes the Dallas/Fort Worth metroplex and Houston, is home to nearly half the residents of Texas. The population within this watershed is expected to double by 2050 [34]. Commercial and recreational fishing on Galveston Bay generates over one billion dollars annually [35]. The areas around the bay are home to some of the nation’s largest petrochemical and industrial complexes; nearly half of all U.S. petrochemical production occurs in the greater Houston area. Further, it is home to three major Ports (Houston, Texas City, and Galveston) which handle upwards of 8000 ships annually (average from 2005–2010), discharging > 10^8^ metric tons of ballast water directly into the bay [18–19]. The actual numbers and volume may be higher since this only reflects vessels reporting. Point (industrial, municipal) and non-point (agricultural, urban, suburban, and rural) discharges are major pollution sources [23,35]. These have been linked to fish kills over the decades, with Thronson and Quigg [16] identifying this bay as a hot spot in Texas. The Galveston Bay Estuary Program (one of 28 EPA National Estuary Programs in the US), identified *beneficial freshwater inflows* as a priority area in its comprehensive conservation management action plan, considering they ‘support the salinity, nutrient, and sediment loading regime needed to ensure the welfare of economically important and ecologically characteristic species in Galveston Bay [35].

The objective of this study was to develop an understanding of the downstream ecological influences of freshwater inflows on water quality and phytoplankton (biomass, community composition) spatio-temporal patterns. Building on the findings of Pinckney [23] and Roelke et al. [24], we used multivariate community analysis methods to assess the phytoplankton variability as correlated to environmental parameters. Given that freshwater inflows have differential influences on the phytoplankton communities of Galveston Bay, alterations upstream (magnitude, timing, frequency) will have a profound effect on downstream ecological processes.

## Materials and Methods

### Ethics Statement

No specific permissions were required for these locations/activities as we were collecting water samples on public waters. There was no animal research or other activities requiring any kinds of permits.

### Study site

**Fig 1:**
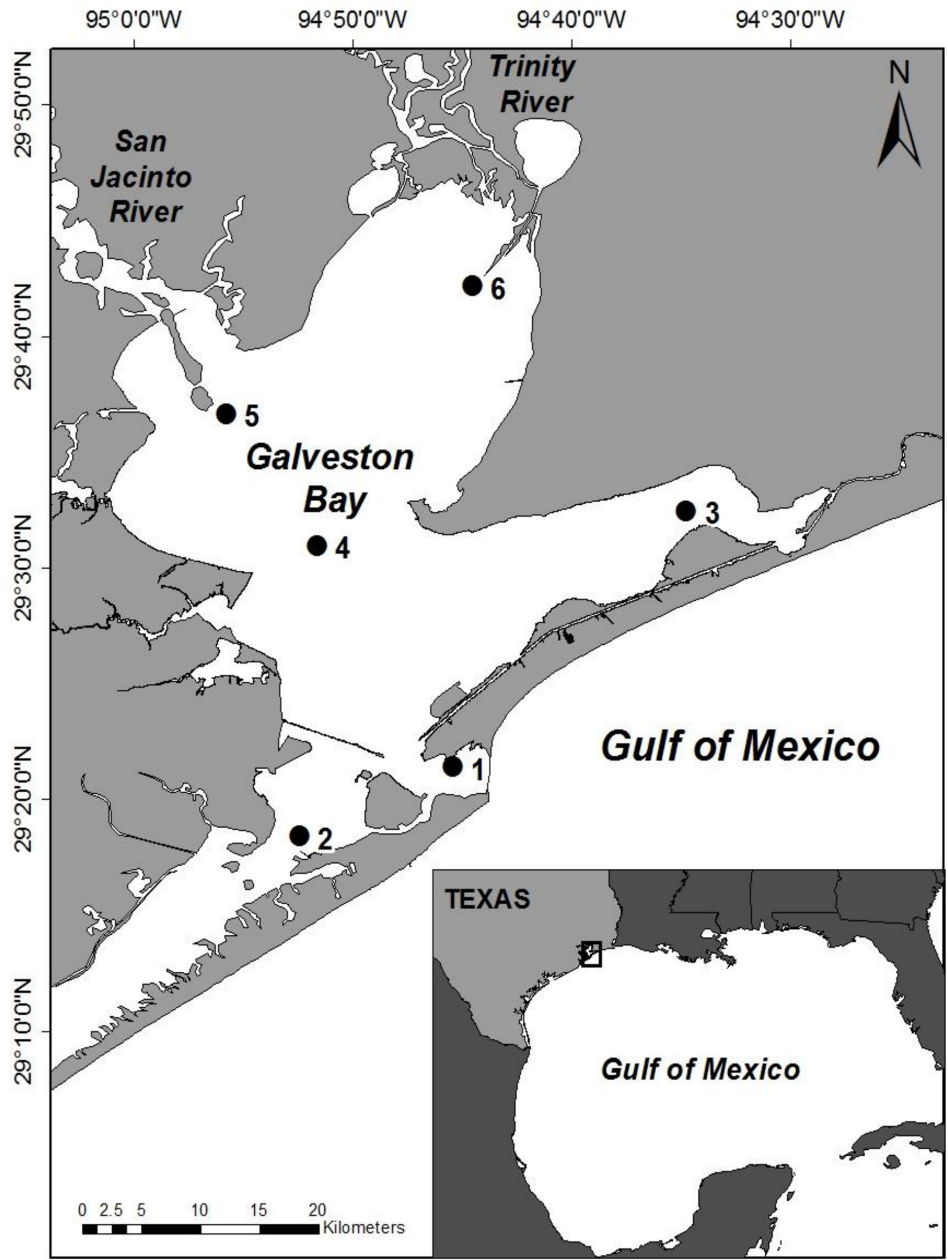
Map of Galveston Bay (Texas, USA) showing the locations of the six fixed stations, the major sources of freshwater inflow – Trinity and San Jacinto Rivers – and the site of the exchange with the Gulf of Mexico (Station 1). Stations are located in the four major sub-Bays: San Jacinto Bay (Station 5), Trinity Bay (Station 6), East Bay (Station 3), and West Bay (Station 2) with Station 4 located in the middle of the Bay.

Galveston Bay, also referred to as the Trinity-San Jacinto Estuary (29.5°N, 94.8°W), is composed of four major sub-bays: San Jacinto Bay (Stn. 5), Trinity Bay (Stn. 6), East Bay (Stn. 3), and West Bay (Stn. 2) (Fig 1). It functions as the drainage basin for the largest watershed in Texas (85,470 km^2^). Guthrie et al. [36] updated surface inflows for the nine rivers, creeks, and bayous draining into Galveston Bay for 1977–2005. Based on the historical hydrologic record, the Trinity River (55%) is the largest provider, followed by the San Jacinto River (16%) and Buffalo Bayou (12%), with significantly smaller contributions from the remaining sub-watersheds (total– 17%). Indirect exchange with coastal water from the Gulf of Mexico occurs primarily through a narrow pass separating Galveston Island and Bolivar Peninsula (Station 1). The tidal regime is microtidal (0.15–0.5 m) such that wind forcing and freshwater inflows are important mixing mechanisms in this shallow bay (~2.1 m average). Daily river flow from the Trinity River was compiled from gauged data (US Geological Survey, Romayor Station #08066500) located ~48 km upriver and above the tidally influenced salt wedge. A monthly average (discharge) and the maximum value for each month (max discharge) were used for the statistical analyses. Rainfall data were obtained from Station Anahuac 6E, Wallisville, near the mouth of the Trinity River (weathersource.com). Fig 1 was prepared using the software ESRI ArcGIS Version 10.2.

### Sampling

Monthly sampling (February 2008 to December 2009) was performed from a small vessel (R/V *Phyto I*). Surface (0–0.5 m) samples collected in acid-cleaned dark polycarbonate bottles from six fixed stations (Fig 1) were stored in an insulated cooler for transportation to the lab (6–8 h; due to size of bay). Nutrient analyses were conducted on water samples filtered under low vacuum pressure (< 130 kPa) through pre-combusted Whatman GF/F filters and the filtrate frozen (-20°C) immediately in acid cleaned high density polyethylene bottles previously triple rinsed with extra filtrate. Nitrate (NO_3_^-^), nitrite (NO_2_^-^), ammonium (NH4^+^), and inorganic phosphorus (DIP) were analyzed according to procedures in [24]. Dissolved inorganic nitrogen (DIN) concentrations were calculated by summing NO_3_^-^, NO_2_^-^, and NH_4_^+^. The detection limit for NH_4_^+^ was 0.070 μmol L^-1^; any values below detection were considered 0.069 μmol L^-1^ for the calculation of DIN. NO_x_ is the sum of NO_3_^-^ + NO_2_^-^; NO_x_ was never below detection. The detection limit for DIP was 0.030 μmol L^-1^; any values below detection were considered 0.029 μmol L^-1^ for calculation of DIN:DIP ratios. Total nitrogen (TN) and total phosphorus (TP) analysis were performed on unfiltered samples according to [24]. The filter from the nutrient analysis was dried at 60°C until a constant weight was reached to determine the total suspended solid (TSS) concentration.

A Fluorescence Induction and Relaxation (FIRe) system was used to calculate the quantum yield for photochemistry (F_v_/F_m_) in surface waters after performing a blank correction [15]. The concentration of chlorophyll (chl) *a* and phytoplankton accessory pigments were measured using HPLC according to Pinckney et al. [37] on samples filtered and immediately frozen (-80°C). Pigment peaks were identified by comparing retention times and absorption spectra with pure standards (DHI, Denmark). The synthetic carotenoid β-apo-8’-carotenal was used as an internal standard. Accessory pigments provide reliable measures of the relative abundance of characteristic algal groups, including fucoxanthin for diatoms, alloxanthin for cryptophytes, peridinin for dinoflagellates, chl *b* for green algae, and zeaxanthin for cyanobacteria [37–39]. Traditional microscopic methods were used to verify that the dominant groups were indeed present as indicated by the HPLC analysis.

### Statistical analysis

Averages are presented plus or minus standard deviation (±SD). Variation in the concentration and relative abundance of accessory pigments was assessed using multivariate community analysis methods using the R computing environment (Version 2.9.0) [40] and a suite of packages, mainly the “vegan” library (Version 1.15–3) [41]. Environmental data were log(x+1) transformed to scale data before subjecting it to any analysis. To calculate the *relative pigment abundance*, a community data matrix was developed using Eqs 1 and 2 in which each accessory pigment concentration was divided by the respective chl *a* concentration (standardization to maximum) for that station:

$$xAP_{1,2,\ldots n}/xCh1_{1,2,\ldots n}=x{'}_{1,2,\ldots n},$$ (1)

and then divided by the sum of all the accessory pigments (standardize to total):

$$x{'}_{1,2,\ldots n}/\sum x{'}_{1,2,\ldots n},$$ (2)

where xAP is the concentration of an accessory pigment (μg L^-1^) and xChl is the concentration of chl *a* (μg L^-1^) in Eqs 1 and 2 respectively. The result is equal to the fraction of each accessory pigment in relation to 1 chl *a* molecule (see also [38]) and is equivalent to Wisconsin double standardization commonly used in ecological community analysis methods [41]. This pigment matrix was used to determine the relative importance of algal groups using environmental vector fitting.

Triangular similarity matrices of pigment concentration and relative abundance data were calculated using the Bray-Curtis index in order to characterize samples according to their similarity in pigment profiles. This pigment concentration matrix was then subjected to hierarchical cluster analysis using Ward’s minimum variance method [42] and a similarity profile test (SIMPROF) was performed using the “clustsig” package in R [43]. SIMPROF allowed us to identify whether the clusters were significantly different from each other based on *p* < 0.05. Averages of the concentration and relative abundance of accessory pigments within each of the clusters was then calculated allowing us to classify groupings according to quantitative and qualitative differences in pigments. The frequency of each month and station between clusters was then assessed to summarize temporal and spatial trends within the sample groupings. Assessing the relative frequency of month and stations among clusters allowed us to assign samples to a season (Winter/Spring, Summer/Fall, and Transitional) and area (North Bay, Mid Bay, East Bay and South Bay).

We then used permutational multivariate analysis of variance (PERMANOVA) [44] to identify whether the overall pigment concentration and relative abundance data were significantly different between designated areas and seasons. We separated the phytoplankton data and ran PERMANOVA for each individual pigment which served as a univariate counterpart to this test. We repeated this to pinpoint the specific pigments whose concentration and relative abundance were significantly different between clusters. To relate these findings back to the environment, we used PERMANOVA to assess whether environmental parameters were significantly different across seasons, areas, and clusters.

We then further evaluated the relationship between the pigment and environmental data as well as functional community parameters including biomass and photosynthetic efficiency (F_v_/F_m_) using the Mantel test of matrix correlation [45,46]. To visualize the spatial autocorrelation, an inter-sample Euclidean distance matrix was constructed for the environmental data and plotted against the dissimilarity matrices of the concentration and relative abundance of accessory pigments. By running a Mantel test against these matrices, a Spearman’s correlation coefficient (*ρ*) was calculated and significance (*p* < 0.05) of the correlation was tested by running random permutations of the data. To assess spatial and temporal dissimilarity, month and station averages were calculated for the pigment and environmental data and their respective dissimilarity matrices were also subjected to Mantel tests. We then ran the Mantel test across all environmental variables to gain a more detailed understanding of the correlation between the phytoplankton and environmental dissimilarity.

Pigment concentration and relative pigment abundance data were then ordinated using non-metric multidimensional scaling (nMDS) following the metaMDS procedure in the “vegan” library [41] to produce two separate ordination maps. An unconstrained ecological interpretation of the ordinations was then performed using environmental vector fitting (envfit procedure) which allowed us to correlate the two dimensions of the nMDS solution to all the environmental parameters collected. Environmental parameters which were significantly correlated to the pigment dissimilarity matrices according to Mantel test were expressed as vectors on the nMDS plot. The length of each vector is referred to as its strength and longer vectors indicate a greater degree of correlation. Stations on the ordination map were color coded according to the cluster they were assigned to when subjected to hierarchical cluster analysis. In addition, station symbols were coded according to a respective season which was determined according to results from calculating the frequency of months within each major cluster.

## Results

### Seasonality of freshwater inflows and water quality

**Fig 2:**
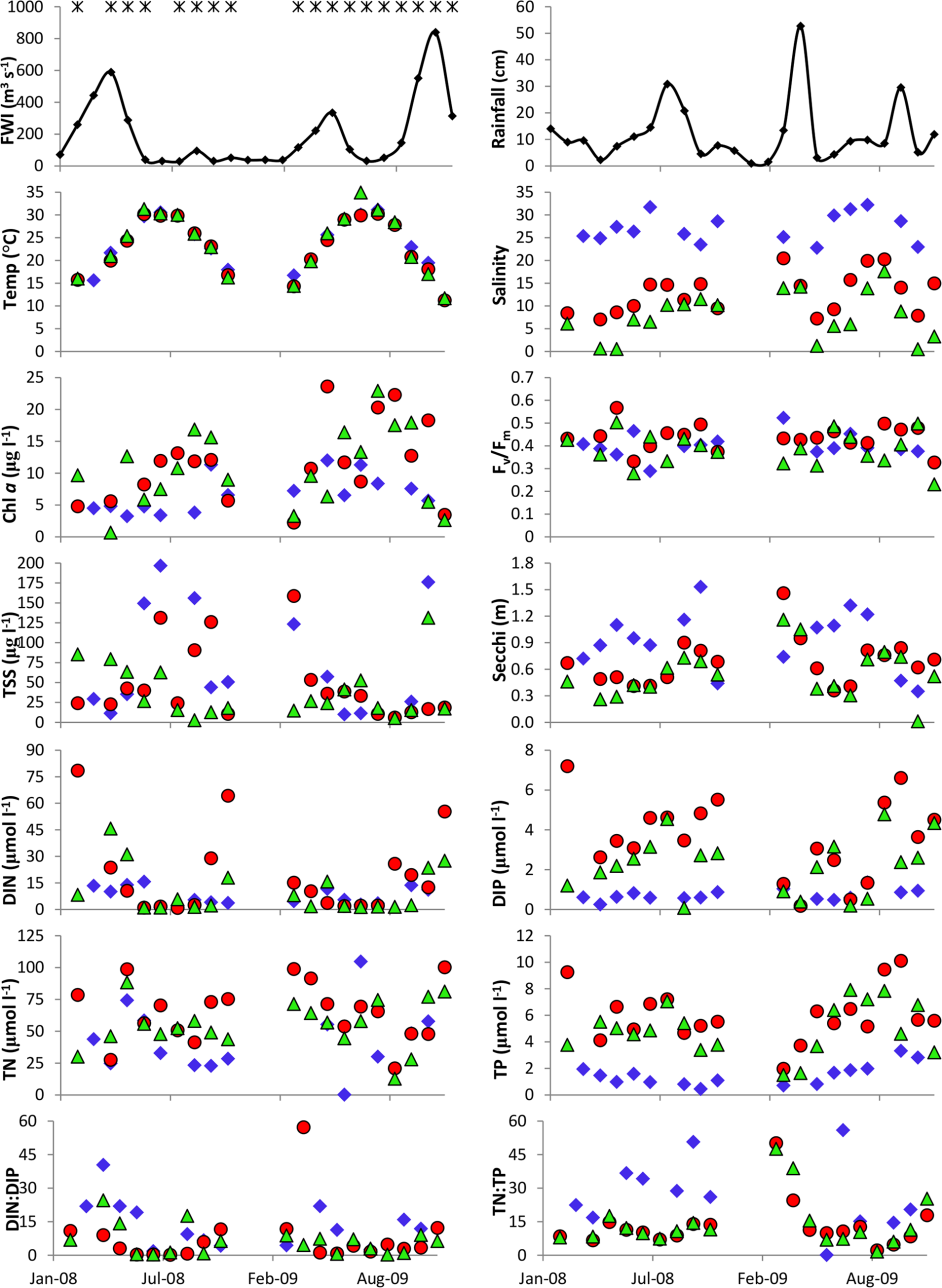
Water quality parameters measured in Galveston Bay during the 2008–2009 sampling campaigns (timing shown in A with *): freshwater inflows on the Trinity River (m^3^ s^-1^; A), rainfall (cm; B), temperature (°C; C), salinity (unitless; D), chl *a* (μg L^-1^; E), F_v_/F_m_ (relative units; F), total suspended solids (μg L^-1^; G), secchi depth (m; H), and nutrient concentrations (µmol L^-1^): DIN (I), DIP (J), TN (K), TP (L), which were then used to calculate ratios of DIN:DIP (M) and TN:TP (N). A subset of the data was used to show important spatial and temporal patterns as well as the ranges of values measured: Station 6 (adjacent to the Trinity River; green triangles), Station 5 (adjacent to the San Jacinto River; red circles) and Station 1 (closest to the Gulf of Mexico; blue diamonds).

Monthly sampling campaigns in 2008/2009 covered both high (typically spring or fall) and low (summer) freshwater inflow events (Fig 2A). Samples were collected in 19 of the 24 months (Fig 2A); when sampling campaigns were missed it was typically due to high winds which frequent the bay especially during winter/spring. There were >30% more freshwater inflows from the Trinity River into the bay in 2009 relative to 2008. Significant freshwater inflow events (freshets) are defined herein as >283 m^3^ s^-1^ [47]. Monthly averaged flows which contributed to freshets were observed from March to May 2008 (spring), March 2009 (spring) and October to December 2009 (fall/winter). There was one false inflow event during the study period recorded immediately after Hurricane Ike made landfall on September 13th 2008; this peak reflects that drainage of surge waters back into Galveston Bay. In 2009, large monthly rainfall events (>25 cm) preceded large inflow events (Fig 2B) while in 2008 this was not the case. Temperatures were warm (October to April) and cool (May to September) (Fig 2C) and with averages of 28.6 °C (±2.5) and 18.2 °C (±3.3) respectively, characteristic of non-arid subtropical systems [47–48].

To observe spatially sensitive as well as temporal patterns in water quality in Galveston Bay, we plotted data measured at Station 6 (adjacent to the Trinity River), Station 5 (adjacent to the San Jacinto River) and Station 1 (closest to the Gulf of Mexico). Although both Stations 5 and 6 directly receive riverine inputs, the water quality is known to be very different and so we show data collected from both these stations. Stations 2 and 3 had very similar patterns to Station 1 while Station 4 located mid-bay, had water quality parameters which were within the ranges experienced at Stations 6, 5 and 1. Salinity (presented on the unit-less practical scale) was lowest in the upper bay (Stations 5 and 6) and highest near its mouth (Station 1; Fig 2D) reflecting the estuarine gradient. Chl *a* did not vary significantly (*p* = 0.15) on temporal scales at Station 1 (Fig 2E; Table 1), with values ranging from 3.24–12 μg L^-1^. During 2008 at Station 5, chl *a* concentrations were lower during winter/spring compared to summer/fall, opposite to inflow patterns. In 2009, they essentially mirrored inflow patterns with only a few exceptions (Fig 2E). Chl *a* concentrations were similar in magnitude (from 0.66 to 23 μg L^-1^) at Stations 5 and 6, but the patterns at each station were very different.

**Table 1:** Results of permutational multivariate analysis of variance and univariate contrasts for pigment concentration (measured), relative pigment abundance (calculated, see methods), and the environmental data performed for (A) season, (B) area and (C) cluster). Significant differences between the phytoplankton and environmental parameters in each of these categories are bold. Non-significant relationships are denoted using n.s and *p*-values for significant results are indicated as follows:

Variables	(A) Season	(B) Area	(C) Cluster
Pigment concentration			
Fucoxanthin	**0.10 (** *** **)**	**0.22 (** *** **)**	**0.20 (** *** **)**
Peridinin	0.01 (n.s.)	0.02 (n.s.)	0.02 (n.s.)
Alloxanthin	0.01 (n.s.)	**0.09 (** ** **)**	**0.07 (** ** **)**
Zeaxanthin	**0.15 (** *** **)**	**0.05 (** * **)**	**0.29 (** *** **)**
Chlorophyll *b*	**0.10 (** ** **)**	**0.04 (** * **)**	0.01 (n.s.)
Relative pigment abundance			
Fucoxanthin	0.01 (n.s.)	**0.23 (** *** **)**	**0.20 (** *** **)**
Peridinin	0.02 (n.s.)	0.02 (n.s.)	0.02 (n.s.)
Alloxanthin	**0.06 (** ** **)**	**0.09 (** ** **)**	**0.07 (** ** **)**
Zeaxanthin	**0.10 (** *** **)**	**0.05 (** * **)**	**0.29 (** *** **)**
Chlorophyll *b*	0.01 (n.s.)	**0.04 (** * **)**	0.01 (n.s.)
Environmental data			
Temperature (°C)	**0.34 (** *** **)**	0.01 (n.s.)	**0.36 (** *** **)**
Salinity	0.01 (n. s.)	**0.33 (** *** **)**	0.01 (n.s.)
F_v_/F_m_	0.01 (n.s)	0.01 (n.s.)	0.01 (n.s.)
NO_x_ (μmol L^-1^)	**0.06 (** * **)**	**0.04 (** * **)**	**0.24 (** *** **)**
NH_4_ (μmol L^-1^)	**0.10 (** ** **)**	0.01 (n.s.)	**0.17 (** *** **)**
DIP (µmol L^-1^)	0.01 (n.s.)	**0.24 (** *** **)**	0.01 (n.s.)
TN (μmol L^-1^)	0.01 (n.s.)	**0.05 (** * **)**	0.01 (n.s.)
TP (μmol L^-1^)	0.01 (n.s.)	**0.55 (** *** **)**	**0.08 (** ** **)**
TSS (mg L^-1^)	0.01 (n.s.)	**0.06 (** *** **)**	0.02 (n.s.)
Secchi (m)	0.01 (n.s.)	**0.06 (** * **)**	0.01 (n.s.)
Chl *a* (μg L^-1^)	**0.15 (** *** **)**	0.01 (n.s.)	**0.38 (** *** **)**
DIN (μmol L^-1^)	**0.06 (** ** **)**	0.04 (0.06)	**0.24 (** *** **)**
Avg. Discharge (m^3^ s^-1^)	0.02 (n.s.)	-	**0.14 (** *** **)**
Max Discharge (m^3^ s^-1^)	0.03 (0.06)	-	**0.12 (** *** **)**

*** p ≤ 0.001

** *p* ≤ 0.01

* *p* ≤ 0.05. For relationships that approached significance (0.05 > p > 0.10), *p*-values are given instead of notations.

There were no trends in F_v_/F_m_ measurements (often used as a proxy of phytoplankton health) (Fig 2F); F_v_/F_m_ varied from 0.23–0.57 with a mean of 0.42 (±0.06). Total suspended solids were highly variable on both spatial and temporal scales at all stations varying >70-fold from 2.67 to 197 μg L^-1^ between 2008 and 2009 (Fig 2G). Secchi depths were generally lower at Stations 5 and 6 which mimicked each other (0.25–1.24 m; mean = 0.62 m) while those at Station 1 were generally higher (0.35–1.53 m; mean = 0.93 m) (Fig 2H). Station 4, in the middle of the bay, had secchi depths from 0.39 to 1.32 m with a mean of 0.68 m. These generally shallow Secchi depths are reflective of the particulate inputs by the two major rivers and the wind driven mixing in this shallow (2.1 m) estuary. The gradient (based on means) reflects the estuarine nature of Galveston Bay.

High freshwater discharge corresponded to increased DIN concentrations (Fig 2I) but not always DIP (Fig 2J). DIN concentrations were always < 20 μmol L^-1^ at Station 1 but as high as 80 μmol L^-1^ at Stations 5 and 6 at different times of the year. The exception was during summer when inflows were generally very low. DIP concentrations were on average 3 times lower in the lower part of the bay compared to the upper riverine influenced stations and less variable (0.72 μmol L^-1^ ±0.26 at Station 1 compared with 2.92 μmol L^-1^ ±1.79 at Stations 5 and 6). While TN concentrations were highly variable on spatial and temporal scales (Fig 2K), TP followed the same general patterns as DIP (Fig 2L). DIN:DIP ratios were generally lower than Redfield’s ratio (8.72±10.38; range 2–57) (Fig 2M). DIN:DIP ratios were on average lower at Station 5 (7.51) and Station 6 (6.36) than at Station 1 (13.23) respectively. TN:TP ratios were similar at Stations 5 and 6 but generally < 16 while those at Station 1 were more variable and higher (Fig 2N).

**Fig 3:**
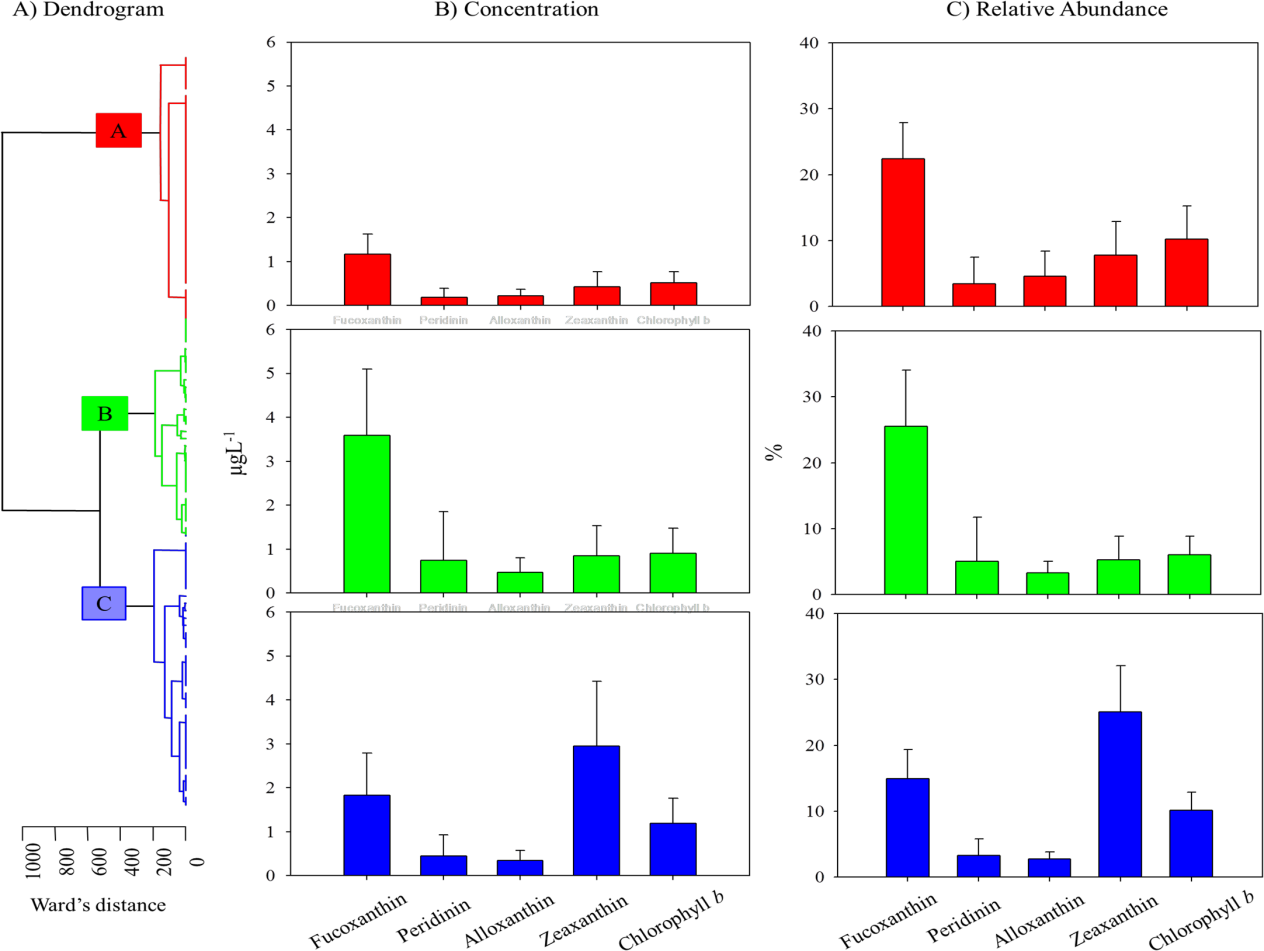
Classification of samples using (A) agglomerative cluster analysis identified clusters which were significantly different from each other. Small clusters originating from same major branch were grouped together (to increase *n* values and statistical robustness) targeting major shifts in phytoplankton community structure. The major clusters are represented throughout as A (red), B (blue) and C (green). To identify differences in the structure of each major cluster, the (B) average pigment concentration and (C) relative pigment abundance (means ± standard deviation) were also calculated.

PERMANOVA and its univariate contrast were used to identify particular water quality parameters as being significantly different across (1) seasons (Winter/Spring, Summer/Fall, and Transitional), (2) areas (North Bay, Mid Bay, East Bay and South Bay) and/or (3) clusters (see Fig 3 below) as summarized in Table 1. With regards to the measured environmental parameters, temperature, NO_X_, NH_4_, DIN, and phytoplankton biomass were significantly different (*p* < 0.05) between seasons (Table 1). Salinity, NO_x_, TN, P_i_, TP, TSS, and secchi depth measurements were significantly different (*p* < 0.05) between areas of the bay. Although the PERMANOVA did not identify freshwater discharge as being significantly different between the designated categorical variables of season, area or cluster (see more below), variation in nutrients, salinity, and turbidity ultimately stem from variation in the timing and magnitude of freshwater inflows.

### Variation in phytoplankton community structure

Cluster analysis of pigments from the 19 sampling campaigns identified six clusters (A, B_1_, B_2_, C_1_, C_2_, and C_3_) that were significantly different from each other (*p* ≤ 0.05) (Fig 3A). These clusters, based on relative concentrations of fucoxanthin, alloxanthin, peridinin, chl *b* and zeaxanthin, ultimately reflect shifts in phytoplankton community structure. We grouped clusters according to their association to one another on the dendrogram (i.e. cluster B consisted of B_1_ and B_2_) to increase the n value and robustness of statistical analyses, and simultaneously targeting the most dominant changes in phytoplankton community. Overall, cluster A had lower pigment concentrations when compared to clusters B and C. Fucoxanthin concentrations (1.17 ±0.45 μg L^-1^) were more than double zeaxanthin concentrations (0.43 ±0.34 μg L^-1^) and greater than all the other accessory pigments (peridinin = 0.18 ±0.21 μg L^-1^, alloxanthin = 0.22 ±0.15 μg L^-1^, chl *b* = 0.51 ±0.25 μg L^-1^) in cluster A (Fig 3B). Stations from cluster B were characterized by the greatest fucoxanthin (3.59 ±1.50 μg L^-1^), peridinin (0.75 ±1.10 μg L^-1^) and alloxanthin (0.47 ±0.32 μg L^-1^) concentrations among the three clusters and exhibited moderate concentrations of zeaxanthin (0.84 ±0.69 μg L^-1^) and chl *b* (0.91 ±0.57 μg L^-1^) (Fig 3B). This suggests a dominance of eukaryotic phytoplankton such as diatoms, dinoflagellates, and cryptophytes. Cluster C exhibited the inverse trend to cluster B with concentrations of zeaxanthin (2.95 ±1.47 μg L^-1^) eclipsing those of the other pigments (fucoxanthin = 1.82 ±0.96 μg L^-1^, peridinin = 0.44 ±0.48 μg L^-1^, alloxanthin = 0.35 ±0.22 μg L^-1^, chl *b* = 1.19 ±0.58 μg L^-1^), signifying cyanobacterial dominance (Fig 3B). This is supported by relative abundance data (Fig 3C), which shows the community composition of clusters A and B was dominated by fucoxanthin (20–25%) while zeaxanthin (25%) was most abundant in cluster C. Results indicate separation between the major clusters stem from quantitative and qualitative differences in the phytoplankton community composition.

### Temporal and spatial trends among clusters

**Fig 4:**
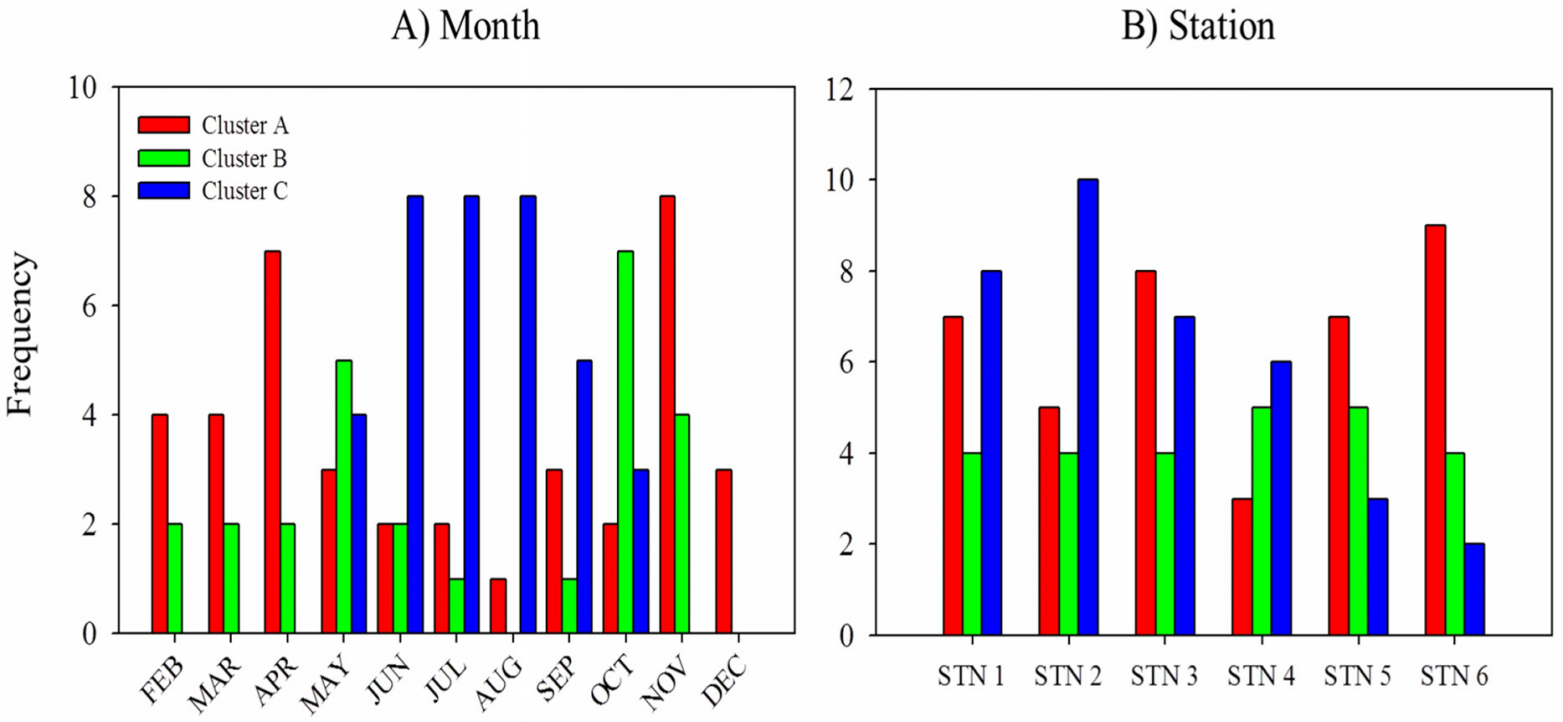
To understand the variation driving the *a posteriori* clustering of samples, we assessed the frequency of samples taken during specific *a priori* categories including months (A) and at specific stations (B) within each of the clusters (see Fig 3). Major clusters are represented as A (red), B (blue) and C (green).

To gain an understanding of the variation driving the classification of samples across time, we assessed the frequency of samples taken during certain months within each of the clusters (Fig 4A). Our analyses show that clusters A and B were dominated by samples taken from November to April while cluster C samples were primarily from June to September. The months of May and October are unique in that they are associated with many of the samples from each of the clusters; these months also serve as transitional periods between warm and cool periods (Fig 2C), respectively. When the relative frequency of these seasonal periods within each cluster is calculated (Table 2), we found that 72.2% of the samples occurring from November to April (winter/spring) were found in Cluster A, 70.7% of the samples from June to September (summer/fall) were found in cluster C and 50.0% of samples from May and October were in cluster B (transitional). These findings suggest that temporal variability strongly influenced the grouping of samples during our study period.

**Table 2:** The relative frequency of *a priori* categories “season” and “area” within *a posteriori* derived hierarchical clusters (see Fig 3A). These factors targeted expected temporal and spatial variability in phytoplankton community structure.

	Cluster A	Cluster B	Cluster C
Season (%)			
Winter/Spring	72.2	27.8	0
Transitional	20.8	50.0	29.2
Summer/Fall	19.5	9.8	70.7
Area (%)			
South Bay (1,2)	31.6	21.1	47.4
East Bay (3)	21.4	35.7	42.9
Mid Bay (4)	42.1	21.1	36.8
North Bay (5,6)	53.3	30.0	16.7

When the frequency of each station was taken into consideration, we were able to establish spatial differences among the phytoplankton community in Galveston Bay (Fig 4B; Table 2). The distribution of pigments in Stations 5 and 6 (North Bay), are similar and a majority of these samples fell in cluster A (53.3%). The northern areas of the bay exhibited low overall phytoplankton biomass with a community composition dominated by diatoms and other eukaryotic phytoplankton. Conversely, an increased number of samples in cluster C originate from Stations 1 and 2 found in the southern part of the bay (47.4%) representing an overall increase in the concentration and relative abundance of cyanobacteria in this area. Stations 3 and 4 are not similar to each other but each is comprised of a pigment profile that is different from the northern and southern stations (Table 2). Station 4 has relatively proportional amount of samples from cluster A and C (42.1 and 36.8%, respectively); this is consistent with its location in the middle of the bay and suggests this is a transitional area where marine water originating from the Gulf of Mexico and fresh water coming from the Trinity and San Jacinto Rivers mix. Station 3 is located in East Bay and likely exhibits a unique pigment profile because it is hydrographically distinct from the rest of the bay.

### Phytoplankton community structure and environmental gradients

PERMANOVA and its univariate contrast were also used to identify particular pigments as being significantly different across (1) seasons, (2) areas and/or (3) clusters as summarized in Table 1. We found the concentration and relative abundance of the pigments fucoxanthin, alloxanthin, and zeaxanthin were significantly different (*p* < 0.05) across these three categorical variables (season, area, cluster) indicating phytoplankton groups containing these pigments within the overall community are the most sensitive to variations in time and space.

**Fig 5:**
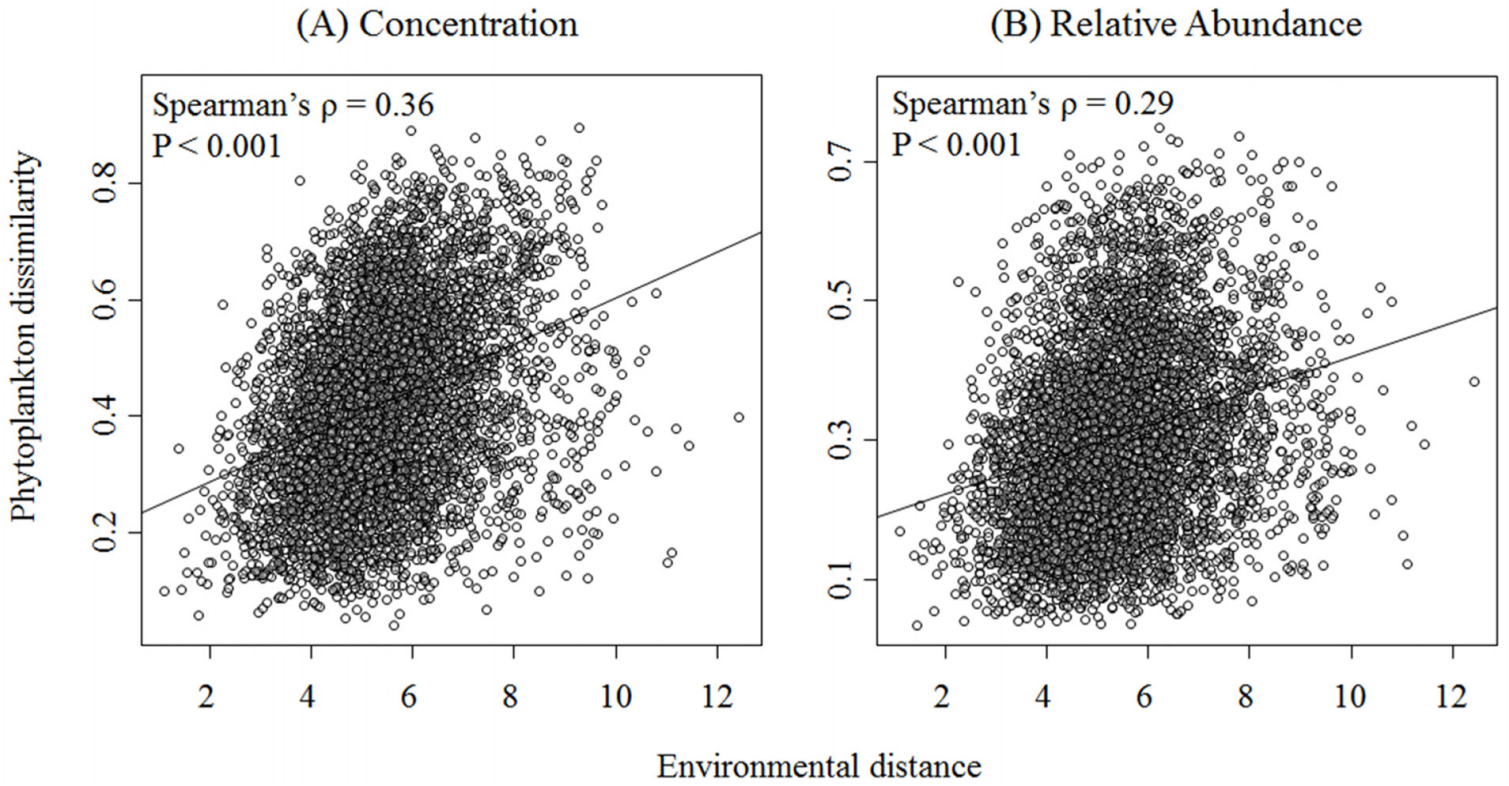
Spearman’s correlation among environmental distances and (A) pigment concentration and (B) relative pigment abundance dissimilarity matrices. Environmental distances represent Euclidean distance, and pigment dissimilarity was calculated using the Bray-Curtis index. Correlation and significance of correlation among matrices was assessed using the Mantel test.

To further investigate the relationship between the environmental variables and phytoplankton in Galveston Bay, we used the Mantel test to directly correlate their respective dissimilarity matrices. Individual pigment concentration and relative pigment abundances were significantly correlated (Spearman’s *ρ* values = 0.36 and 0.29, respectively; *p* < 0.001) to environmental distances representing the overall environmental conditions (i.e., the combination of all 16 individual parameters) (Fig 5A and 5B respectively). This suggests that differences in the phytoplankton community structure increased along changing environmental gradients. To pinpoint the environmental properties that had the greatest influence on phytoplankton communities, we assessed the correlation of single environmental variable distances to the phytoplankton dissimilarities (Table 3). Our results show the dissimilarity in pigment concentrations was significantly (*p* < 0.05) related to differences in temperature (*ρ* = 0.28), F_v_/F_m_ (*ρ* = 0.18), NO_x_ (*ρ* = 0.18), NH_4_^+^ (*ρ* = 0.18), TP (*ρ* = 0.07), overall biomass (chl *a*, *ρ* = 0.71), DIN (*ρ* = 0.20), average freshwater discharge (*ρ* = 0.11) and maximum freshwater discharge (*ρ* = 0.11). When the relative abundance of pigments was tested, significant correlations (*p* < 0.05) included temperature (*ρ* = 0.30), salinity (*ρ* = 0.08), F_v_/F_m_ (*ρ* = 0.14), NO_x_ (*ρ* = 0.11), NH_4_^+^ (*ρ* = 0.10), P_i_ (*ρ* = 0.05), TP (*ρ* = 0.11), overall biomass (*ρ* = 0.15), DIN (*ρ* = 0.10), average freshwater discharge (*ρ* = 0.23) and maximum freshwater discharge (*ρ* = 0.24). Properties of freshwater inflow were found to correlate to differences in phytoplankton biomass and community composition which supports hierarchical cluster analysis and PERMANOVA results (Table 3).

**Table 3:** Spearman’s correlation (*ρ*) matrix between pigment concentration and relative pigment abundance dissimilarities and environmental distances (total and individual) tested for significance using Mantel test. Significant correlations are bolded, non-significant correlations are denoted using n.s., and *p*-values for significant results are indicated as follows:

	Pigment concentration	Relative pigment abundance
Latitude	0.02 (n.s.)	0.01 (n.s.)
Longitude	-0.02 (n.s.)	-0.01 (n.s)
Temperature (°C)	**0.28 (** *** **)**	**0.30 (** *** **)**
Salinity	0.05 (n.s.)	**0.08 (** * **)**
F_v_/F_m_	**0.18 (** *** **)**	**0.14 (** *** **)**
NO_x_ (μmol L^-1^)	**0.18 (** *** **)**	**0.11 (** *** **)**
NH_4_ (µmol L^-1^)	**0.18 (** *** **)**	**0.10 (** ** **)**
DIP (μmol L^-1^)	0.01 (n.s.)	**0.05 (** * **)**
TN (μmol L^-1^)	-0.02 (n.s.)	-0.03 (n.s.)
TP (μmol L^-1^)	**0.07 (** * **)**	**0.11 (** ** **)**
TSS (mg L^-1^)	0.05 (n.s.)	0.04 (n.s.)
Secchi (m)	0.01 (n.s.)	0.01 (n.s.)
Chl *a* (μg L^-1^)	**0.71 (** *** **)**	**0.15 (** ** **)**
DIN (μmol L^-1^)	**0.20 (** *** **)**	**0.10 (** * **)**
Avg. Discharge (m^3^ s^-1^)	**0.11 (** *** **)**	**0.23 (** *** **)**
Max Discharge (m^3^ s^-1^)	**0.11 (** *** **)**	**0.24 (** *** **)**

***: *p* ≤ 0.001

** *p* ≤ 0.01

* *p* ≤ 0.05

**Fig 6:**
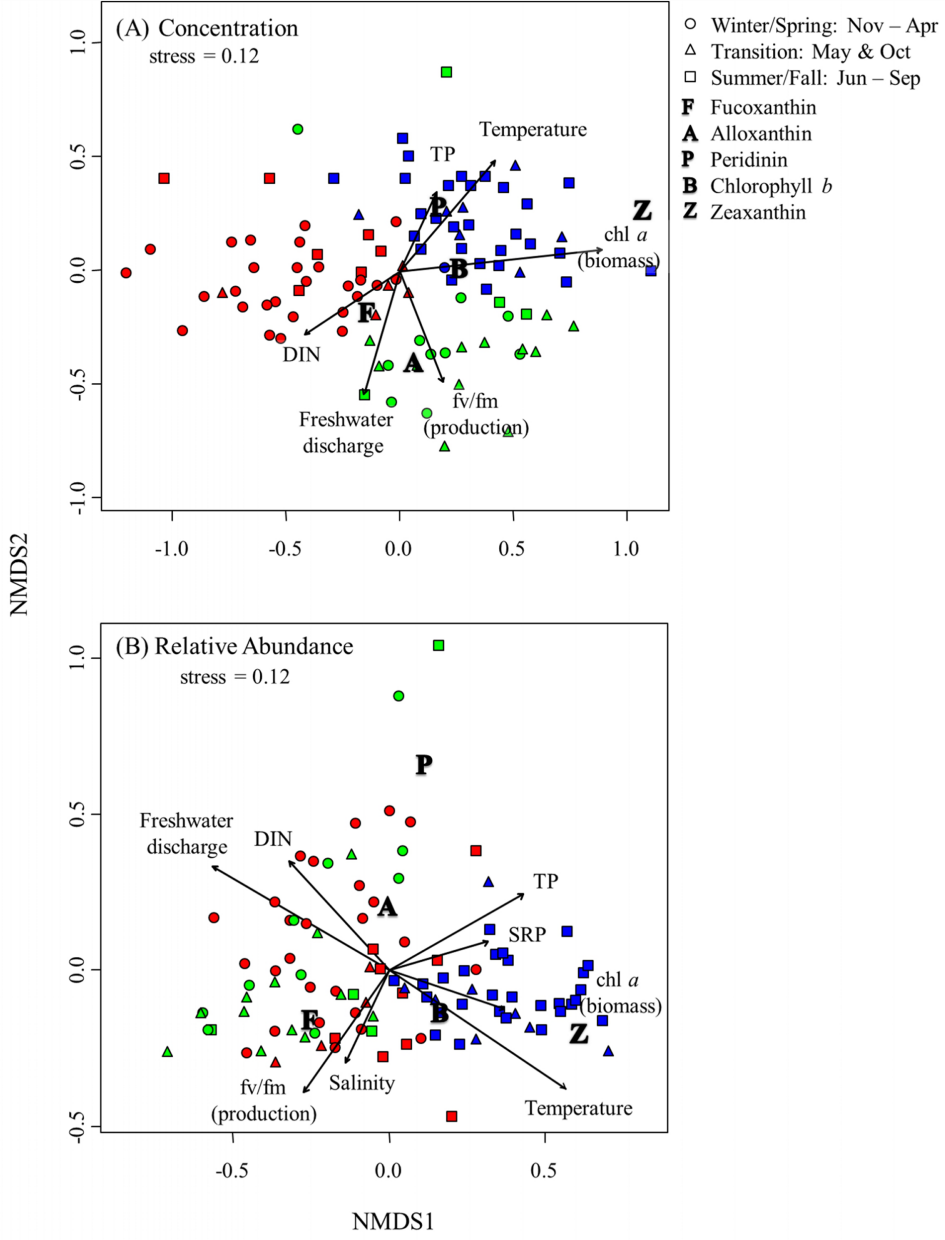
Similarity in (A) pigment concentration and (B) relative pigment abundance visualized in ordination space and coded according to results of the agglomerative cluster analysis (in Fig 3) showing Cluster A (red), B (blue) and C (green). Seasons are defined as winter/spring (November-April, ○) and summer/fall (June-September, □) with two transitional months (May and October, ∆). Phytoplankton pigments included are fucoxanthin (F) for diatoms, alloxanthin (A) for cryptophytes, peridinin (P) for dinoflagellates, chl *b* (B) for green algae, and zeaxanthin (Z) for cyanobacteria. Non-metric multidimensional scaling coupled to environmental vector fitting shows significant environmental variables that correlate to pigment data as vectors on the plot.

To illustrate the interactions within our data set, we visualized the sample data in ordination space (nMDS) and projected environmental variables as vectors on the plot using environmental vector fitting. For individual pigment concentrations, we found samples from cluster A occurred on the negative side of nMDS axis 1 while those from clusters B and C lie on the positive side of this axis (Fig 6A). Dissimilarity between clusters B and C is also evident with samples from cluster B separated from cluster C along the second nMDS axis. Differences in season are also visible in Fig 6A: separation between winter/spring and summer/fall months is strong along nMDS axis 1 (circles vs. squares). The separation among transitional months (May and October) occurs on both the positive and negative side of nMDS axis 1 but samples are relatively concentrated in the second quadrant of the graph (+nMDS 1,-nMDS 2). The strength and gradient of freshwater discharge (DIS) and DIN vectors indicate that samples in the spring/winter (cluster A) exhibited decreased overall phytoplankton biomass. Samples taken during summer months exhibited overall greater zeaxanthin concentrations and accompanied by increased temperature and TP measurements (cluster C). Furthermore, Cluster B was composed mainly of transitional months (May and October; summer/fall) and increased diatom pigments and F_v_/F_m_. The relationship between pigment concentrations and the environment is difficult to discern because they all occur on the positive side of NMDS axis 1.

When relative pigment abundance data were subjected to the same analysis; we found significant overlap between clusters A and B but not C (Fig 6B). This suggests that community composition between clusters A and B is similar and characterized by an increase in the relative abundance of fucoxanthin, supporting results of hierarchical cluster analysis (Fig 3C). On the other hand, Cluster C lies on the positive side of the first nMDS axis and its relationship to increased concentrations of zeaxanthin is shown (Fig 6B). Again, there is a clear separation between winter/spring months and summer/fall months with transitional periods distributed evenly across the first nMDS axis. There appears to be a gradient from fucoxanthin to zeaxanthin that is less apparent when pigment concentration data is subjected to nMDS analysis (Fig 6A). This may also stem from the overall low concentrations of all accessory pigments in cluster A. In addition it appears that some samples were characterized by increased peridinin concentrations indicating a short term increase in the level of this pigment (e.g., as a result of a dinoflagellate bloom). The strength and gradient of freshwater discharge, DIN, salinity and F_v_/F_m_ vectors indicates these parameters were increased in samples characterized by increased relative abundance of fucoxanthin, peridinin and alloxanthin pigments. The vector properties of temperature, chl *a*, P_i_, and TP indicate that these parameters were higher at stations that were dominated by cyanobacteria and green algae.

## Discussion

Worldwide, water regulators are faced with meeting demands of rising human populations, while maintaining critical riverine inputs of freshwater to sustain healthy estuaries and coasts, and their valuable ecosystem services [2–6,10–11]. Along with concerns of ongoing anthropogenic nutrient enrichment and human modification on hydrologic and hydrographic regimes, more recently, the impact of climate change is being considered [20–21]. Three factors are key and arguably the most important. First, droughts are predicted to increase in frequency and severity, and subsequently reduce freshwater delivery to estuaries [20,48–49]. Second, flooding may enhance nutrient loading, erosion and mobilization of land based nutrients [48,50]. Third, in most of the world’s oceans phytoplankton biomass has declined dramatically over the past century [51] while estuarine chl *a* has generally been found to be increasing [5,13,52]; both these have long term implications for fisheries. While the influence of freshwater inflows on salinity, nutrients, and turbidity in Texas estuaries have been generally described [23–24,50,53], the effects on biological components, especially phytoplankton, are less understood.

### Spatial patterns

Recently Roelke et al. [24] defined for the first time co-occurring and opposing freshwater inflow effects on phytoplankton biomass, productivity and community composition in Galveston Bay for the period 2005–2006. In the sampling area adjacent to the San Jacinto River (equal to Station 5, Fig 1), inflows stimulated phytoplankton biomass and productivity while the opposite pattern was seen in the region near the Trinity River (equal to Station 6, Fig 1). Phytoplankton were frequently nutrient limited, responding rapidly to nutrient loading from the San Jacinto River while hydraulic displacement (and perhaps other processes such as grazing) resulted in overall lower phytoplankton biomass in the Trinity River delta. Given average inflows from the Trinity River (1977–2005) are ~3.5 times greater than the San Jacinto River [36], significantly greater riverine inflows negated the effect of nutrient loading on the phytoplankton in the upper northeastern sector of Galveston Bay.

Following these arguments to another station located in the mid the bay region (Station 4), it appears that phytoplankton respond to both nutrients and hydraulic activity, with both magnitude and duration of an inflow event defining which factor is more important at any one time. In the lower regions of Galveston Bay (Stations 1–3), phytoplankton biomass, productivity and nutrient concentrations are generally lower and not predictable or correlated with salinity and temperature as previously observed [24,27]; these stations are more reflective of the higher-salinity-nutrient-poorer waters frequently exchanged with the Gulf of Mexico. We observed similar such patterns in terms of biomass and productivity (not shown). Not unexpectedly, freshwater inflows have a diminishing influence on phytoplankton biomass with distance from the source. This phenomenon has been observed in other estuaries [39,54–57]. The present study and Roelke et al. [24] however, add that the behavior of the source (i.e., magnitude of flows) has important consequences and multiplicative effects, including co-occurring and opposing, that are influencing the phytoplankton community.

Stations 5 and 6 closest to riverine inputs (Trinity and San Jacinto Rivers) for Galveston Bay were characterized by greater biomass of diatoms and diversity of phytoplankton groups (diatoms, dinoflagellates, cryptophytes, green algae) compared to Stations (1–3) in the southern part of the bay. The latter were characterized by low overall phytoplankton biomass dominated by primarily cyanobacteria. Örnólfsdóttir et al. [58] found pulsed N input events preferentially favored increases in diatom biomass in the Trinity River delta (near our Station 6), and could even induce a shift from initially cyanobacteria dominated community to one of diatoms. Roelke et al. [24] found that cyanobacteria, haptophytes and euglenophytes did not respond to river inflows on the San Jacinto River side of the bay to the same level as diatoms. It was postulated that stimulation of these groups may not be driven by nutrient loading alone as they are able to utilize a variety of alternative nutrient acquisition strategies (i.e., diazotrophy, mixotrophy) not typically employed by diatoms. Alternatively, these groups are more sensitive to hydraulic displacement given their overall slower growth rates. Our observations of high diatom biomass in upper Galveston Bay despite the higher flows from the Trinity River compared to the San Jacinto River reflects environmental conditions, their overall faster growth rates and/or that the displacement of this phytoplankton group by inflows is low compared to their growth rates. The multivariate statistical analyses show that phytoplankton responses (biomass and community composition) are directly tied to parameters associated with freshwater inflow such as discharge (average and maximum) from the Trinity River, as well as DIN (and NO_X_, NH_4_) concentrations. These environmental parameters were found to be significantly different (*p* < 0.05) between sample assemblages identified using hierarchical clustering and significantly correlated (*p* < 0.05) to phytoplankton dissimilarity matrices. Considering the calculated flushing rates for Galveston Bay (0.08–0.035 d^-1^; [24]) are significantly lower than diatom growth rates (1–2 d^-1^), the ability of diatoms to accumulate in the upper region of the bay relative to other groups appears to be the result of their inherent physiological characteristics.

In the subtropical coastal Indian River Lagoon (Florida, USA), Badylak and Phlips [59] found that dinoflagellates, diatoms and cyanobacteria were the significant contributing phytoplankton groups to this system. It maybe that the low tidal mixing energy present there that is more favorable to dinoflagellates, which while present in Galveston Bay (present study; [18–19,58,60]) are not as abundant. It appears that dinoflagellates and cyanobacteria are favored the mid-and lower regions of Galveston Bay based on the multivariate analyses; locations where neither hydraulic displacement nor nutrient loading from freshwater inflows would have been important, but where relatively high residence times and low tidal mixing energy may contribute to their standing crops. Motile phytoplankton such as dinoflagellates and some species of cyanobacteria are selectively favored where vertical mixing energy is limited, partly because of their ability to access alternative carbon and nitrogen resources [61–62]. Chan and Hamilton [54] found diatoms, dinoflagellates and green algae were the main groups in the Swan River estuary, Western Australia, strongly separated spatially by flow and salinity (and temporally by season). As with the current study, diatoms occurred under a wide range of inflows while dinoflagellates were present only at very low discharges (this was attributed to group specific differences in potential growth rates). Unlike Galveston Bay, chlorophytes (green algae) were found to be important by Chan and Hamilton [54], but restricted to freshwater conditions. In the Swan River estuary, nutrients were found to be less important than flow and salinity in regulating phytoplankton biomass and succession while in Galveston Bay, nutrients were equally as important. The findings of Choudhury and Pal [56] for the Bhagirathi–Hooghly estuary in India are similar to those of Chan and Hamilton [54] in terms of primary driving factors and phytoplankton patterns. As in these two studies, Quigg et al. [63] found the switch between green algae and cyanobacteria in Moreton Bay (northeastern Australia) could mostly be explained by gradients in water clarity/turbidity. Hence, the magnitude of freshwater inflows relative to the physical properties of an estuary (e.g., size, depth) appear to be important in defining which groups of phytoplankton respond most strongly, and may explain why green algae were less important in Galveston Bay compared to other estuarine systems.

### Temporal patterns

Although we highlight the role of freshwater inflows as a driver of phytoplankton structure, we must not overlook the importance of temperature (seasons) and other key variables in defining temporal patterns. Differences in temperature during the 2008–2009 sampling period correlated well with differences in the concentration and relative concentrations of pigments representing phytoplankton communities in Galveston Bay. Cluster analysis showed that winter and spring months are characterized by increased diatom, dinoflagellate and cryptophyte abundance while cyanobacterial abundance was higher during summer months in Galveston Bay, consistent with earlier studies [23,23,60] and in other estuaries [39,57]. This is partially driven by phytoplankton metabolism, with growth optima in the range 15–18°C and 20–23°C for diatoms and dinoflagellates respectively, and with cyanobacteria having higher growth optima in the 25–30°C range [6].

Months characterized by the dominance of diatoms (winter/spring) corresponded to periods of higher freshwater discharge and DIN concentrations. The dominance of diatoms among the phytoplankton community subsided during the summer months when freshwater discharge was also low. It can be rationalized from our findings that during summer months, a decrease in the magnitude of freshwater inflows leads to nutrient limitation, most likely as nitrogen, of the diatom community. At the same time, cyanobacteria which are able to more efficiently utilize nutrients because of their size and potential for diazotrophy, may outcompete the diatoms. Although the extent of N_2_ fixation in Galveston Bay has yet to be established, this deduction is supported by DIN measurements which show that inorganic nitrogen was scarce during summer/fall months and abundant during winter/spring months. These characteristics have been employed in management strategies to reduce the prevalence of cyanobacterial blooms [26].

Previous studies using short term bioassays in Galveston Bay found the resident phytoplankton community to be stimulated by nitrogen [23,58,60], specifically diatoms whose growth rate was enhanced by nitrate additions. It has also been argued that phosphorus also plays a role [47]. Benthic phosphorus regeneration at the end of summer is a recurring process in the Galveston Bay estuary [23,27]; the impact on the phytoplankton community may be due to relief of summertime P_i_ limitation. Recent studies reveal that co-limitation by both nitrogen as nitrate and P_i_ is more commonly measured than either N (as nitrate) or P limitation alone in this [47] and other coastal systems [29,30,63].

Light limitation is known to be an important regulator of phytoplankton in estuaries and coastal systems [28,30–31,47,63], but did not appear to be an important factor in structuring the phytoplankton community in Galveston Bay in 2008 and 2009 or previously. Örnólfsdóttir et al. [60] found light limitation to be negligible in this system due to wind driven vertical mixing which keeps the shallow bay well mixed. We found that secchi depth and TSS were significantly different between areas of the bay but these factors ultimately did not control phytoplankton community structure (Tables 1 and 3).

### Transitional periods

Further research is needed to study the variables contributing to the succession of phytoplankton groups in Galveston Bay. Herein we identified May and October as key transitional periods where the foremost phytoplankton groups (diatoms and cyanobacteria) shift in dominance. Both historically and during the study period (2008–2009), May and the months leading up to it are characterized by the period of greatest freshwater inflow into Galveston Bay [23,47,53]. The diatom dominated spring bloom and subsequent collapse during late spring appears to be linked to changes in riverine inputs and may be important to the succession of cyanobacteria during the summer. Choudhury and Pal [56] also observed significant switches in the dominant phytoplankton groups (and diversity indices) timed with the summer/winter transition. Arguably, consequences of the key climate change factors being considered (drought, flooding) are going to be felt most strongly during these transitional periods, particularly in terms of implications for water quality, fisheries and health. For example, in a meta-analysis of water quality in the Dickinson Bayou watershed, a subsystem of Galveston Bay, Quigg et al. [48] identified shifts to elevated bacteria (fecal coliform, *Escherichia coli* and *Enterococcus*) and depressed dissolved oxygen concentrations (<3 mg l^-1^) during the warmer months when the water column stratified, and then a switch back in the cooler months. In years when the timing of these events was altered, fish kill events were more likely to be observed.

### Food web implications

Although the details regarding trophic relationships in Galveston Bay have yet to be thoroughly understood, it is recognized that freshwater inflows play a major role in structuring food webs in this estuary [35]. For example, Buyukates and Roelke [64] assessed the interactions between the phytoplankton and zooplankton community structure. It was found that pulsed inflows resulted in increased zooplankton biomass in all treatments which were accompanied by a concurrent decrease in phytoplankton biomass but an increase in species diversity. If freshwater inflows increase the abundance or variety of food sources for consumers in estuarine environments, it is reasonable to assume that the changes in the timing and magnitude of freshwater inflow events will alter the biomass of consumers and food web structure in those environments.

Grazing of phytoplankton by the benthic community in Galveston Bay may be more important than the grazing impacts associated with zooplankton. Örnólfsdóttir et al. [60] reported that grazers were responsible for consuming as much as 75% of daily phytoplankton production in the bay. The role of benthic communities in regulating phytoplankton may also explain some of the spatial patterns observed. For example, the Eastern oyster (*Crassostrea virginica*) found in the mid-region of the bay, is responsible for an estimated one-third of the state’s fishing income [35,65]. While it is understood that alterations in freshwater inflows influence the fecundity of oysters (quantity of spat as well as disease and parasite levels) [65]; less is known about the role of phytoplankton (biomass, community composition) (Sammy Ray, pers. comm).

Freshwater inflows have also been shown to contribute positively to the abundance and diversity of intertidal and benthic floral and faunal species assemblages in other Texas estuaries [20,23,35,47,50,53,55], but freshwater interactions among these assemblages in Galveston Bay have yet to be established. Identifying interactions between freshwater inflows and salt marsh communities is also critical as they serve as nursery habitats for important consumer organisms in this estuary. Assessing the stable isotopic abundance of carbon (δ^13^C) and nitrogen (δ^15^N) has been used to describe trophic relationships in estuarine food webs [38]. In addition, δ^15^N has been used to trace anthropogenic nutrient inputs to marine systems and their eventual incorporation into food webs [38]. Applying this powerful method to trace freshwater inflows and discern trophic relationships between food webs in Galveston Bay will undoubtedly increase our understanding of how structure and function in estuaries is driven by freshwater inflow events.

In Texas estuaries and others worldwide, scientists, engineers and resource managers have been challenged to define processes and develop freshwater management strategies which balance understanding of the current status of ecosystems against past changes and future pressures, studies such as the present one are important towards developing an understanding of the downstream ecological processes which are influenced by freshwater inflows.
